# CyberKnife Radiosurgery in Recurrent Brain Metastases: Do the Benefits Outweigh the Risks?

**DOI:** 10.7759/cureus.3741

**Published:** 2018-12-17

**Authors:** Romagna Alexander, Christoph Schwartz, Barbara Ladisich, Wolfgang Hitzl, Sarah-Charlotta Heidorn, Peter A Winkler, Alexander Muacevic

**Affiliations:** 1 Neurosurgery, Christian-Doppler-Medical Center, Paracelsus Private Medical University, Salzburg, AUT; 2 Biostatistics, Christian-Doppler-Medical Center, Paracelsus Private Medical University, Salzburg, AUT; 3 Medical Physicist, European CyberKnife Center Munich, Munich, DEU; 4 Radiosurgery, European CyberKnife Center Munich, Munich, DEU

**Keywords:** recurrent brain metastases, local treatment, cyberknife radiosurgery

## Abstract

Introduction

Local treatment concepts are in high demand in the salvage treatment of recurrent brain metastases. Still, their risks and benefits are scarcely characterized. In this study, we analyzed the outcome and risk-/benefit-ratio of salvage CyberKnife (Accuray Incorporated, Sunnyvale, California, US) radiosurgery in the treatment of recurrent brain metastases after whole brain radiotherapy (WBRT).

Materials and methods

Seventy-six patients with 166 recurrent brain metastases and a multimodal pretreatment were retrospectively investigated. All patients underwent salvage CyberKnife radiosurgery (single fraction, reference dose: 17-22 Gy). Study endpoints were post-recurrence survival (PRS) after salvage treatment as well as local and distant tumor control rates. Central nervous system (CNS) toxicity was assessed according to the toxicity criteria of the Radiation Therapy Oncology Group and the European Organization for Research and Treatment of Cancer (RTOG/EORTC)).

Results

The population was homogenous regarding its demographic parameters. All patients had a history of WBRT prior to salvage CyberKnife radiosurgery. PRS was 13.3 months (10.4 - 16.2 months), one-year local and distant tumor control rates were 87% (95% CI: 75-99) and 38% (95% CI: 23-52), respectively. Eighteen patients suffered from RTOG/EORTC grade I/II toxicity. No toxicity-related risk factors were identified.

Discussion

This study found indicative survival and tumor control rates as well as a favorable risk/benefit ratio regarding radiotoxicity in salvage CyberKnife radiosurgery. These results point to a proactive therapeutic strategy based on appropriate patient selection instead of therapeutic nihilism.

## Introduction

The prognosis of patients suffering from recurrent intracranial metastases remains dismal [1-2]. The majority of these patients is diagnosed with progressive brain disease within the first 14 months, with a median time to central nervous system (CNS)-related death of nine months [2]. The lack of standardized treatment algorithms for these patients leads to a variety of individualized salvage treatment strategies [3]. Possible concepts consist of local treatment options (percutaneous radiotherapy/radiosurgery, open tumor resection (OTR), and low-activity iodine-125 brachytherapy (SBT) or systemic chemotherapy and/or any combinations thereof [3-6]. For salvage radiosurgery, a post-recurrence survival (PRS) range between 4.5 and 22.4 months - depending on the tumor entity - has been reported [7-8]. Aside from survival, the matter of safety remains a major concern in the salvage treatment of brain metastases. The oncological impact of salvage radiotherapy has to be balanced against the known risk of radionecrosis and morbidity [4].

In the current study, we analyzed the role of CyberKnife (Accuray Incorporated, Sunnyvale, California, US) radiosurgery for circumscribed recurrent brain metastases as local salvage treatment concepts. The aim of this study was to elucidate the place of this treatment modality with a special focus on treatment-associated morbidity and treatment burden. The hereby provided results may help to further improve the development of local salvage treatment concepts for brain metastases and facilitate future patient selection/counseling.

## Materials and methods

Patient selection

A group of patients suffering from recurrent brain metastases was retrospectively identified. Salvage CyberKnife radiosurgery was conducted at the European Cyberknife Center Munich between August 2005 and July 2017. Individualized treatment decisions were agreed upon by interdisciplinary neurooncological consensus. Common eligibility criteria included patient- and tumor-related covariates: (1) Karnofsky Performance Score (KPS) ≥60 and an estimated survival prognosis of at least three months; (2) circumscribed tumor recurrence with the presence or absence of mass effect on pre-interventional magnetic resonance imaging (MRI); and, ultimately (3) the patient preferences. The maximum contrast-enhancing tumor diameter should not have exceeded 3 cm on preoperative MRI scans. Prior written informed consent was obtained from all patients.

CyberKnife radiosurgery 

For treatment planning, the MRI protocols consisted of T1-weighted ± gadolinium contrast medium, T2-weighted, and fluid-attenuated inversion recovery (FLAIR) sequences, with a slice thickness of 1.0 mm. O-(2-[18 F]fluoroethyl)-1-tyrosine positron emission tomography (FET-PET) was applied with increasing frequency over the course of the treatment, as previously described, but was not part of the standardized management algorithm [9]. Three-dimensional isocentric/conformal noncoplanar treatment planning was routinely chosen to match the tumor volume as previously described. The radiation dose was single-fractioned with a range from 17 to 22 Gy [10].

Clinical and radiological follow-up

The initial clinical and radiological follow-up was routinely planned three months after salvage CyberKnife radiosurgery. Further follow-up evaluations were conducted at three- to six-month intervals, depending on the individual patient’s clinical status. Radiological follow-up was routinely performed by MRI scans. Follow-up FET-PET scans were conducted for selected patients only. All imaging data of the follow-up were reviewed independently by at least two examiners blinded to clinical and histological data. Tumor recurrence/progression after salvage therapy was determined according to the Macdonald criteria [11].

Assessment of the treatment safety

Any treatment-associated symptomatic effects were recorded for all patients. Treatment toxicity was assessed using the Radiation Therapy Oncology Group/European Organization for Research and Treatment of Cancer (RTOG/EORTC) criteria [12]. Peri-interventional steroid treatment was performed in all cases. After one week, steroids were reduced depending on the neurological status [10].

Statistical analysis

Data consistency was checked and data were screened for outliners. The Cox proportional-hazards model was used to estimate local and distant recurrence rate as well as overall survival. To analyze potential risk factors, the Cox proportional-hazards model was used by using the sigma-restricted coding of factors and Breslow’s method for the adjustment of ties. The Cox proportional assumption was tested by using a Chi-square test for each model. In a first step, a set of potential risk factors was tested for significance in univariate models. In the second step, all significant variables were included in a multivariate model and insignificant variables were excluded as long as all variables in the multivariate models remained significant. The Wilcoxon matched pairs test was used to compare continuously distributed variables. All reported tests were two-sided, and p-values < 0.05 were considered as statistically significant. All statistical analyses in this report were performed by using NCSS (NCSS 10, NCSS, LLC. Kaysville, UT, US), Statistica 13 (Hill, T. & Lewicki, P. Statistics: Methods and Applications. StatSoft, Tulsa, OK, US), and PASW 21 (IBM SPSS Statistics for Windows, Version 21.0., Armonk, NY, US) and were done by one of the authors (WH). For this type of retrospective analysis with a mere descriptive design, no formal consent was required.

## Results

Study population

Seventy-six patients with 166 recurrent brain metastases were identified and included for the analysis. The population was homogenous regarding age, KPS, and histology. Detailed demographic data are summarized in Table 1.

**Table 1:** Basic population characteristics

	Total
Number of patients	76
Age (years) median	55.6 (33.6-82.4)
Gender male/female	25 / 51
KPS (pre/post-treatment) median	90/90
Cumulative volume (cm^3^) median	2.8 (0.1-21.8)
CyberKnife dose (Gy) median	18 (16-21)
Primary cancer origin lung / breast / gastrointestinal system / skin / kidney / uterus / other	37 / 25 / 4 / 4 / 3 / 1 / 2

Calculated preoperative cumulative tumor volumes were 2.8 cm^3^ (0.1 - 21. 8 cm3). More than one pre-treated metastasis was seen in 38 cases (two in 17 cases, three in nine cases, four in four cases, five in two cases, six in two cases, seven in three cases, eight in one case). All 76 patients had undergone prior WBRT. The primary treatment protocols consisted in surgery in 16% of patients and in a combination of radio and chemotherapy in 84% of the patients. Median follow-up after salvage CyberKnife radiosurgery was six months (0.6 - 92.5 months).

Outcome

**Figure 1:**
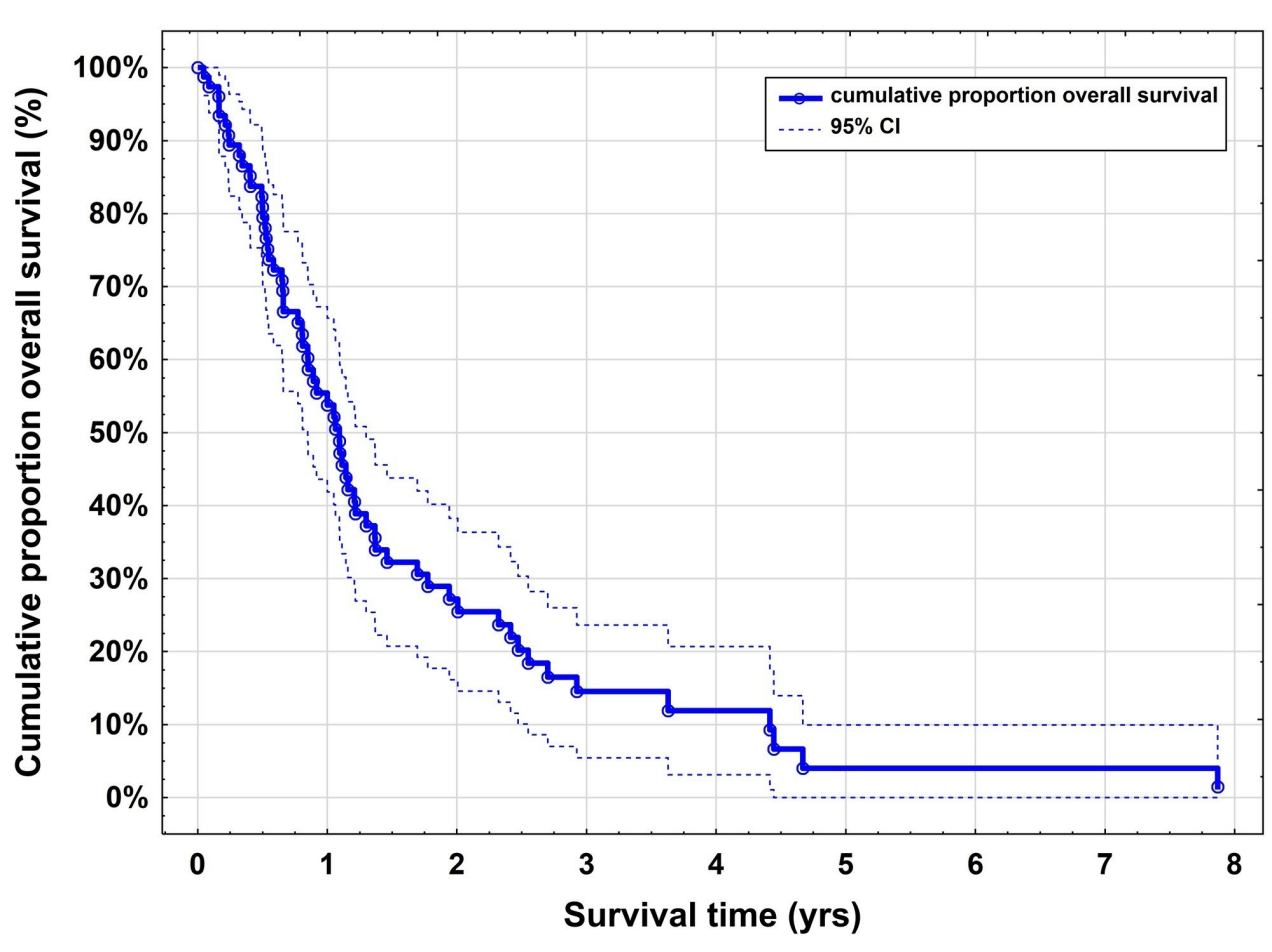
Kaplan-Meier survival curves for recurrent brain metastases after CyberKnife radiosurgery One-year PRS is 54% (95% CI: 42-66) with a median of 13.3 months (10.4 - 16.2 months)

Sixty of 76 patients had died at the time of the last clinical follow-up. Patients succumbed due to progressive extracranial disease in 42% of cases. Median PRS was 13.3 months (10.4 - 16.2 months; Figure 1).

One-year local tumor control rate was 87% (95% CI: 75-99; Figure 2) and the one-year distal tumor control rate was 38% (95% CI: 23-52; Figure 3).

**Figure 2:**
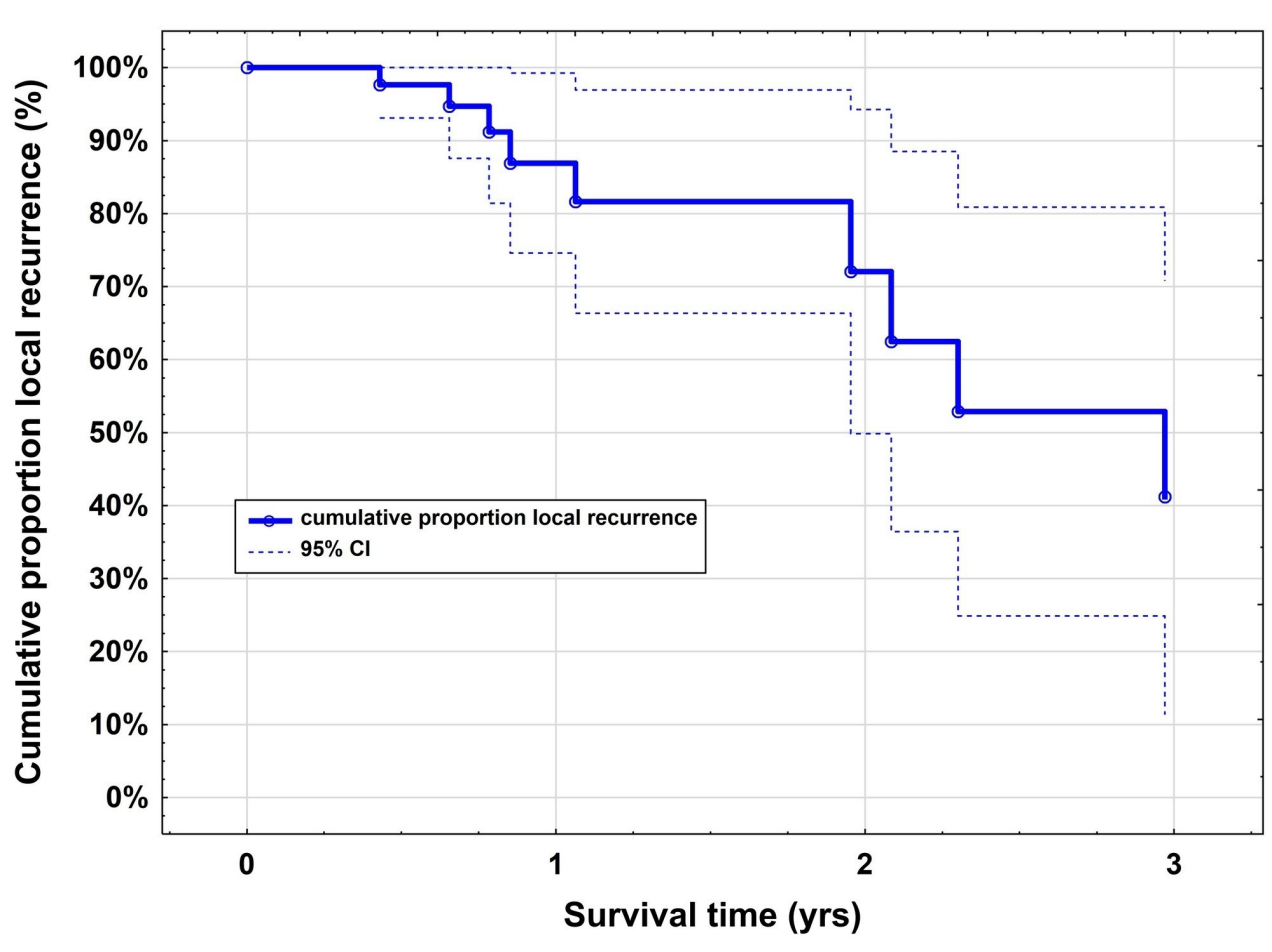
Kaplan-Meier curves for the local tumor control rate after CyberKnife radiosurgery The one-year local tumor control rate was 87% (95% CI: 75-99)

**Figure 3:**
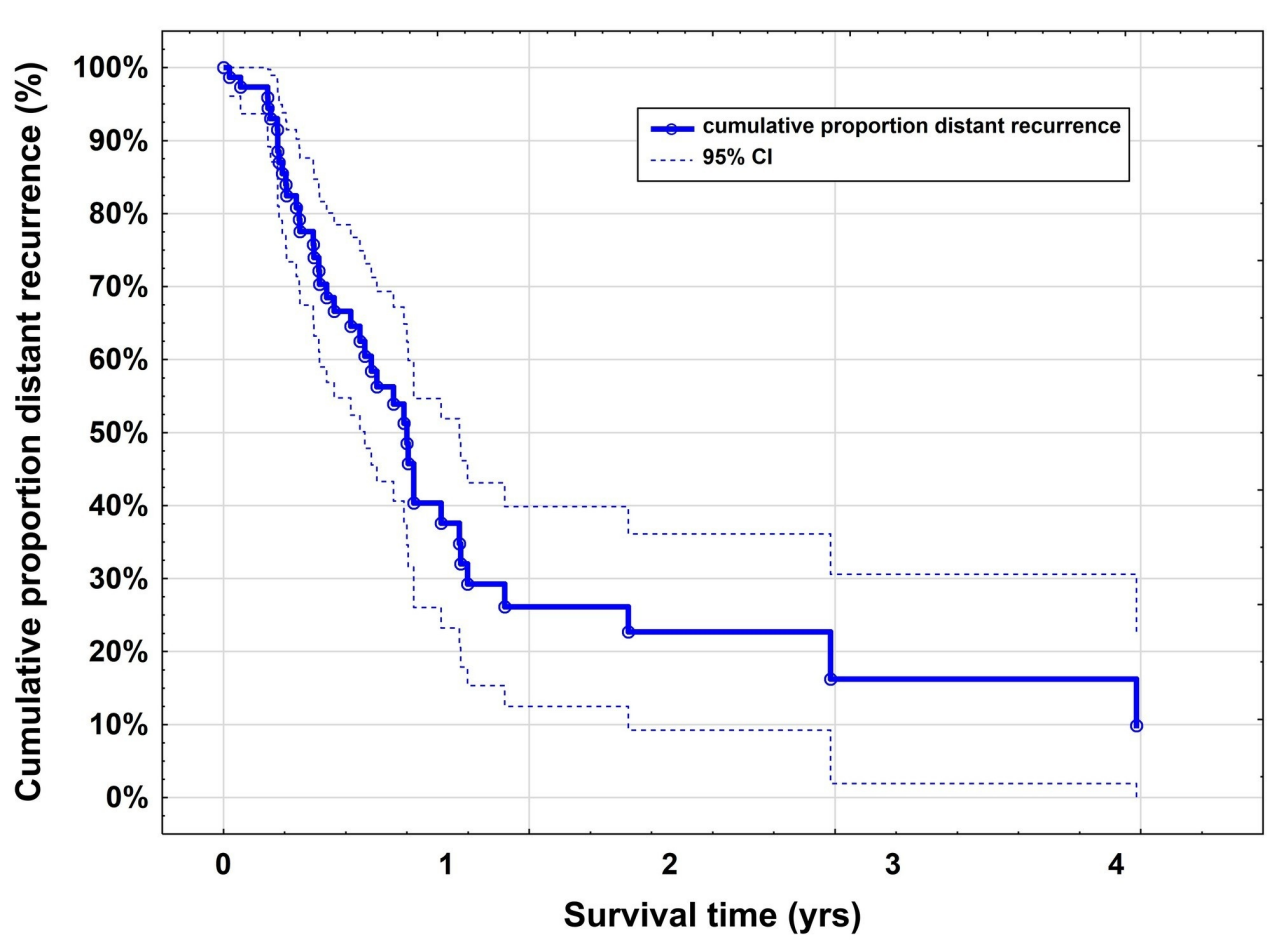
Kaplan-Meier curves for the distant tumor control rate after CyberKnife radiosurgery The one-year distant tumor control rate was 38% (95% CI: 23-52)

Over the course of follow-up, local retreatment was necessary in nine out of 76 patients due to progressive brain disease. The most commonly applied treatment was SBT in 50% of cases. None of the analyzed patient, tumor, and treatment-related factors had a prognostic impact on PRS or tumor control rates in univariate or multivariate models (data not shown).

Treatment toxicity

No treatment-associated mortality was documented and no new permanent neurological deficits were observed. Eighteen patients suffered from transient RTOG/EORTC grade I/II toxicity (i.e. edema), which responded sufficiently to temporary oral steroids. Regarding the length of hospitalization, the treatment was exclusively performed as an outpatient procedure.

## Discussion

Standardized treatment algorithms for patients suffering from recurrent brain metastases are still lacking [13]. Although local treatment concepts represent strategic cornerstones of salvage therapy, the significance of repeat radiosurgery remains a matter of debate. This is mainly due to its potential toxicity [14-16]. This study aimed to provide a detailed analysis of the outcome data, prognostic factors, and toxicity of this treatment modality. Moreover, the study should provide a basis upon which other local salvage treatment concepts, such as OTR, could be compared to. In a mere comparison of survival data, PRS rates after OTR are comparable to our PRS rates after salvage CyberKnife: retrospective data on salvage OTR suggest a PRS range between 7.5 and 12.9 months [4-5]. These ranges are determined by disease- and patient-specific differences. Tumors treated by OTR are more often of larger size, singular, and less commonly located in eloquent areas. Also, the place of both CyberKnife radiosurgery and OTR within the sequence of received salvage treatments has to be kept in mind. For instance, salvage CyberKnife radiosurgery is frequently applied at a later stage of the disease in a more selected population while salvage OTR is sometimes applied in an emergency setting (e.g. for the management of acute hydrocephalus secondary to posterior fossa masses [17]). The comparable survival and tumor control rates apply not only to salvage OTR but also to SBT [6,18]. As previously described, SBT is another valid alternative that combines histological verification and treatment and is applicable in the salvage situation due to low treatment-associated toxicity [6,19]. It was predominantly used in repeated recurrences in our series. Regrettably, these comparable results for local treatment options emphasize our continuing limited effectiveness in the treatment of patients suffering from recurrent brain metastases. Besides the analysis of survival and tumor control rates, an important aspect of this study was to put a focus on the modality’s treatment burden. The treatment burden was subsumed as the treatment-associated morbidity and CNS toxicity according to RTOG/EORTC. Three important issues regarding the aspect of treatment burden have to be kept in mind. First, any additionally caused treatment-associated morbidity may cause a delay or even pose a complete hindrance to the patient’s further adjuvant treatment and might, therefore, subsequently negatively impact survival rates. Second, all patients suffering from recurrent brain disease have already undergone extensive and arduous treatment over the course of their disease and they will eventually succumb to their underlying illness. Thus, the maintenance of an acceptable quality of life for as long as possible - aside from achieving prolonged PRS - has to be regarded as of crucial importance. Hence, keeping the time of treatment-associated hospitalization to a minimum should be considered in each treatment decision. Our CNS toxicity rate of 23.7% is comparably low, given that recent data from the literature indicate a range between 19% and 44% [20]. The third aspect that needs to be considered is the potential postponement of further adjuvant systemic treatment by the performance of local salvage therapy. During CyberKnife radiosurgery, practically no pausing of a systemic treatment is necessary. This is in contrast to OTR or even minimally invasive SBT, where systemic treatment has to be usually discontinued and surgeons might have to face hematologic side effects, such as leucopenia and/or thrombocytopenia [21]. In conclusion, we believe that the neuro-oncological community will have to address the challenge of a steady increase of patients suffering from recurrent brain metastases by implementing standardized salvage treatment concepts. From a strictly neuro-oncological point of view, it is of great interest to improve the selection process of patients who will eventually benefit the most from non-invasive vs. invasive salvage treatment. The treatment burden appears to be minimal for patients undergoing CyberKnife radiosurgery. Undoubtedly, space-occupying, surgically well-accessible tumor recurrences of large volume should be considered for OTR. For patients with single or multiple small tumor recurrences, even in deep-seated and eloquent locations, CyberKnife radiosurgery appears to be the favorable approach. As of now, a careful risk/benefit assessment for each treatment option over the course of the patients’ disease ought to be performed. Future prospective, comparative studies on different salvage treatment options are needed to better define their specific roles in the cascade of available salvage treatments and facilitate patient selection accordingly.

## Conclusions

Standardized salvage treatment concepts for recurrent brain metastases are still lacking and the patients’ PRS remains daunting. In this study, salvage CyberKnife radiosurgery resulted in survival rates that were comparable to values from the literature. Treatment toxicity rates were contained. This renders salvage CyberKnife radiosurgery - in combination with its low treatment burden - an important tool within the neuro-oncological armamentarium (especially in small, deep-seated lesions). Future prospective studies are needed to better define and standardize the cascade of available salvage treatments and facilitate patient selection accordingly.
