# Correction: CyberKnife Radiosurgery in Recurrent Brain Metastases: Do the Benefits Outweigh the Risks?

**DOI:** 10.7759/cureus.c18

**Published:** 2019-03-05

**Authors:** Alexander Romagna, Christoph Schwartz, Barbara Ladisich, Wolfgang Hitzl, Sarah-Charlotta Heidorn, Peter A Winkler, Alexander Muacevic

**Affiliations:** 1 Neurosurgery, Christian-Doppler-Medical Center, Paracelsus Private Medical University, Salzburg, AUT; 2 Neurosurgery, St. Michael's Hospital, University of Toronto, Toronto, Ontario, CAN; 3 Biostatistics, Christian-Doppler-Medical Center, Paracelsus Private Medical University, Salzburg, AUT; 4 Department of Ophthalmology and Optometry, Paracelsus Medical University, Salzburg, AUT; 5 Medical Physicist, European CyberKnife Center Munich, Munich, DEU; 6 Radiosurgery, European CyberKnife Center Munich, Munich, DEU

The first author's name was erroneously entered backwards (Romagna Alexander). The correct name is Alexander Romagna. The manuscript has been updated to reflect this change.

